# Olfactory Dysfunction in Patients with Neuromyelitis Optica

**DOI:** 10.1155/2013/654501

**Published:** 2013-09-25

**Authors:** Felix Schmidt, Önder Göktas, Sven Jarius, Brigitte Wildemann, Klemens Ruprecht, Friedemann Paul, Lutz Harms

**Affiliations:** ^1^Department of Neurology, Charité-Universitätsmedizin Berlin, Charitéplatz 1, 10117 Berlin, Germany; ^2^Clinical and Experimental Multiple Sclerosis Research Center, Charité-Universitätsmedizin Berlin, Berlin, Germany; ^3^Department of Otolaryngology, Charité-Universitätsmedizin Berlin, Berlin, Germany; ^4^Division of Molecular Neuroimmunology, Department of Neurology, University of Heidelberg, Heidelberg, Germany; ^5^NeuroCure Clinical Research Center and Experimental and Clinical Research Center, Charité-Universitätsmedizin Berlin and Max Delbrueck Center for Molecular Medicine, Berlin, Germany

## Abstract

*Background*. Neuromyelitis optica (NMO) is a severely disabling autoimmune disorder of the CNS, which mainly affects the optic nerves and spinal cord. However, recent studies have shown that extra-opticospinal are more common in NMO than previously thought. *Objective*. To investigate olfactory function (OF) in patients with neuromyelitis optica (NMO) versus healthy controls (HC). *Methods*. Psychophysical testing of the orthonasal OF was performed using the *Threshold-Discrimination-Identification* test (*TDI*), measuring different qualities of olfaction, in 10 unselected NMO patients and 10 HC. *Results*. Five of 10 NMO patients (50%) showed hyposmia, while all 10 HC were normosmic. Moreover, NMO patients had significantly lower mean *TDI*-scores compared to HC, based on a poorer performance in both the *Discrimination* and the *Identification* subtests. *Conclusions*. Our results suggest that hyposmia might be part of the expanding clinical spectrum of NMO.

## 1. Introduction

Neuromyelitis optica (NMO, Devic's syndrome) is an autoimmune central nervous system disorder that predominantly affects the optic nerves and the spinal cord [1, 2]. Impaired olfaction is increasingly recognized in neurodegenerative diseases such as Parkinson's and Alzheimer's diseases and has been reported in patients with multiple sclerosis (MS) [3, 4]. As the clinical presentation of NMO may extend beyond relapses of optic neuritis and myelitis [2, 5], we here investigated whether olfactory function (OF) is altered in NMO.

## 2. Patients and Methods

This pilot study was performed from July 2011 to October 2012. Ten patients with NMO according to the 2006 diagnostic criteria [1] were prospectively recruited from the Charité outpatient clinics. Aquaporin-4 antibodies were tested using a commercially available cell-based assay employing recombinant human target antigen (EUROIMMUN, Luebeck, Germany) [6]. A healthy control group (HC) of 10 individuals closely matched for gender and age (±3 years) was recruited among the hospital staff. Exclusion criteria for both groups were olfactory disorders (postinfectious, posttraumatic, and sinunasal), infections of the upper respiratory tract, tumours treated with radiation or chemotherapy, allergies, major depression, and Parkinson's or Alzheimer's disease. All study participants declared to be nonsmokers. Patients taking drugs that could cause olfactory dysfunction as for example amitriptyline and D-penicillamine were excluded from the study. Furthermore, patients receiving corticosteroid treatment during the testing period were excluded because corticosteroids can have an effect on the OF. Olfaction was evaluated using the tripartite *Threshold-Discrimination-Identification test* (*TDI*), based on pen-like odour-dispensing devices as recommended by the “Working Group Olfactology and Gustology” of the German ENT Society [7]. The *Threshold* test (*T*-test) consists of 48 “sniffing sticks” with a 16-stage dilution series of n-butanol for determining the olfactory perception threshold of a patient. The *Discrimination* test (*D*-test) consists of 48 sniffing sticks to test the distinction of different smells. Everyday smells have to be identified with the *Identification* test (*I*-test). Low scores in each subtest demonstrate low olfactory performance. The scores of all three subtests are summed up to the *TDI*-score. A *TDI* value of less than 16 means anosmia, up to 30 points hyposmia, and above 30 points normosmia. Statistical analysis was performed with GraphPad Prism 4.01 (San Diego, USA). The Mann-Whitney *U* test was used to compare medians of olfactory test results between NMO patients and the control group. Proportions of hyposmic patients between both groups were compared by Fisher's exact test. The significance level was defined at *P* < 0.05. Owing to the exploratory nature of this pilot study, no adjustments for multiple testing were made. The study was approved by the ethics committee of the Charité, and all participants gave written informed consent.

To assess the binding of NMO-IgG to AQP4 expressed in the olfactory bulb, 7 *μ*m adult rat and mouse olfactory bulb cryosections (10 *μ*m) were incubated with 10% phosphate-buffered formalin for 4 minutes. After three washes in phosphate-buffered saline (PBS), detergent (1% CHAPS in PBS) was applied for 4 minutes. After three additional washes in PBS, PBS containing 10% normal goat serum was applied for 60 minutes. The sections were then incubated with patient and control sera (1 : 60) for 60 minutes at room temperature. A commercial Alexa Fluor 488-conjugated goat anti-huIgG antibody (Invitrogen, Darmstadt, Germany) was used to detect bound patient IgG. After 60 minutes, the wells were washed thoroughly in PBS, and a glass coverslip was applied to each slide with ProLong Gold mounting medium (Invitrogen).

## 3. Results

Five out of ten patients (50%) with NMO showed hyposmia, none was anosmic. The remaining patients had a normal olfactory function. By contrast, all control subjects were normosmic (Table 1). Two of the hyposmic patients were not aware of their olfactory deficit. Moreover, both mean total *TDI*-scores and *D*- and *I*-subscores were lower in NMO patients compared to HC (Table 1), indicating lower olfactory capacity. The hyposmic NMO patients had a mean *TDI*-score of 26 with a standard deviation of 2.0. Four out of five (80%) hyposmic patients were positive for AQP4 antibodies. The prevalence of olfactory dysfunction was higher in AQP4-IgG-positive patients (67%) than in AQP4-IgG-negative patients (25%), though the difference was not significant. There was no association between OF and disease duration.

**Figure 1:**
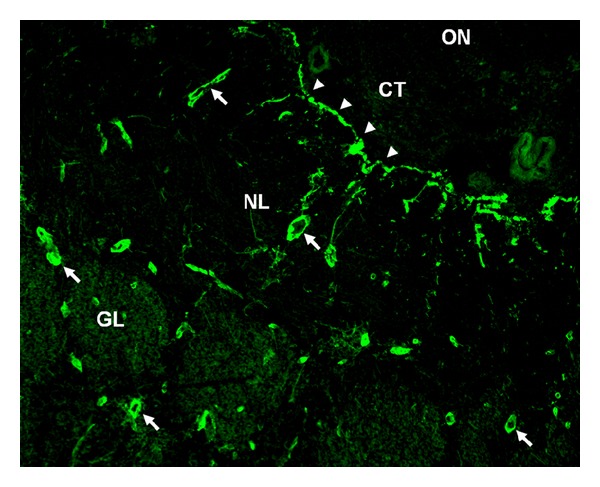
Binding of serum NMO-IgG to the olfactory bulb as demonstrated by indirect immunohistochemistry on formalin-fixed rat tissue cryosections. Binding of the patient's IgG is shown in green (AF488). See method section for details. The figure shows a transverse section through the right olfactory bulb near the medial surface. Arrowheads = pia mater; arrows = microvasculature; GL = olfactory glomeruli; ON = peripheral olfactory nerves (ON); NL = nerve fibre layer; CT = connective tissue next to the medial surface of the olfactory bulb.

**Figure 2:**
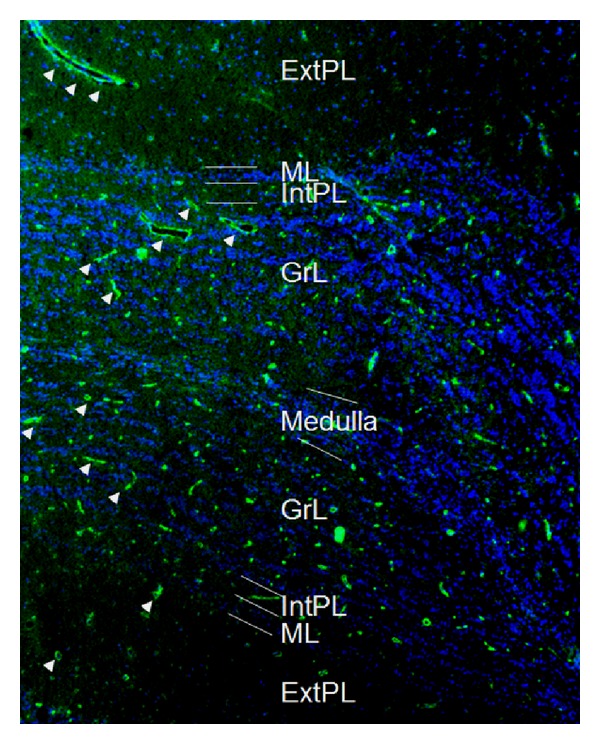
Binding of serum NMO-IgG to the inner portion of the rat olfactory bulb as demonstrated by indirect immunohistochemistry. See results section for details. Binding of the patient's IgG is shown in green (AF488); cell nuclei are shown in blue (4′,6-diamidino-2-phenylindole). Arrowheads = microvasculature; ExtPL-external plexiform layer; ML-mitral cell layer; IntPL = internal plexiform layer; GrL = granular layer.

*Immunohistochemistry.* In accordance with the NMO-IgG staining pattern originally described on cerebellum tissue sections, strong staining of the astrocytic endfeet adjacent to the pia mater and the microvasculature was found (Figures 1 and 2). Only mild, if any, staining of the olfactory glomeruli was detected. No significant binding was present in the peripheral olfactory nerves, the connective tissue next to the medial surface of the olfactory bulb, or (apart from perivascular staining) any of the inner olfactory bulb layers. Overall, twenty tissue sections (10x mouse, 10x rat) were stained. The staining pattern described above was observed with 5/5 unselected AQP4-Ab-positive NMO samples, but not with three unselected AQP4-Ab-negative NMO samples and not with two healthy control samples. Identical patterns were observed when mouse instead of rat tissue sections were employed, though use of rat tissue resulted in a more intense staining (not shown).

## 4. Discussion

Our study revealed orthonasal hyposmia detected by psychophysical testing in five out of ten NMO patients. Four out of five hyposmotic patients were positive for AQP4 antibodies. Our findings are in line with the broadening spectrum of clinical manifestations of NMO that goes far beyond the classical bouts of optic neuritis and myelitis [5] and now encompasses various brain stem symptoms, pain, and the posterior reversible encephalopathy syndrome. 

Impaired olfaction can result from damage to different sections of the olfactory pathway. Peripheral damage is mostly caused by anatomical abnormalities of the nasal cavities, especially the olfactory cleft, and damage to the olfactory epithelium and the olfactory nerve. Olfactory dysfunction can also be a consequence of damage to or atrophy of the olfactory bulb, the olfactory cortex, and higher processing regions of the olfactory system, such as the limbic system. 

In clinical routine, examination of olfactory functions is not regularly performed, presumably owing to the time-consuming procedures required to objectify impaired olfaction. However, olfactory dysfunction has been described in numerous neurodegenerative diseases [3], including MS. Here, olfactory dysfunction results from CNS damage as indicated by an association between olfactory function and lesion load in the olfactory brain [4] and a relation of olfactory functions to longitudinal changes in plaque numbers in central olfactory structures [8]. We could report a relation between the lesion load in the olfactory cortex and the volume of the olfactory bulb and olfactory brain [9]. 

In NMO, which is nowadays considered a disease entity distinct from MS [10], recent neuroimaging studies have consistently reported brain abnormalities in more than 50% of patients [5, 11], however, without particularly investigating lesions or volume reductions in olfactory CNS structures. We appreciate that neuropathological or MRI studies demonstrating typical NMO lesions in olfactory structures would be required to formally prove the supposed link between NMO and OD in our patients. Importantly, however, our results provide a strong rationale for the inclusion of such analyses in future multicentre studies on NMO. 

AQP4, the main target antigen in NMO, is expressed both in the nasal olfactory mucosa including the basal cells, the supporting cells, and the Bowman glands that are part of the olfactory epithelium as a microenvironment necessary for olfaction [12] and in the astrocytic endfeet sealing the microvasculature, and the pial surface of the olfactory bulb; accordingly, we found a strong staining of the olfactory bulb by AQP4-IgG-positive NMO sera (see Figures 1 and 2). AQP4-IgG has been previously shown to cause tissue damage by complement activation and antibody-dependent cell-mediated cytotoxicity [13, 14]. Olfactory dysfunction could thus occur due to tissue damage in olfactory structures with AQP4 expression. Whether mechanisms other than AQP4-IgG were involved in the pathogenesis of hyposmia in the single “seronegative” patient (who otherwise met all diagnostic criteria for NMO) remains unknown. Alternatively, “seronegativity” in this patient could be caused by limited sensitivity of the currently available immunoassays [15]. In myasthenia gravis (MG), another autoimmune disorder with proven humoral pathogenesis, it took almost 25 years until a more sensitive class of immunoassays was developed; subsequently, two-thirds of patients previously classified as “seronegative MG” were found to harbour low-affinity acetylcholine receptor (AChR) serum autoantibodies [16].

In conclusion, our results suggest that hyposmia might be part of the expanding clinical spectrum of NMO and provide the rationale for large-scale, multicentre studies on the frequency of olfactory dysfunction in NMO.

## Figures and Tables

**Table 1:** Demographic data and means of olfactory test results in NMO patients and healthy controls.

	NMO (*N* = 10)	Controls (*N* = 10)	*P* value
Age (years; mean ± SD)	45.1 ± 13.3	46.9 ± 14.49	0.4
Sex ratio (m : f)	1 : 4	1 : 4	1
AQP4 antibody (pos./neg., *N*)	6/4	n.a.	n.a.
Disease duration (months; mean SD)	28.2 ± 43.5	n.a.	n.a.
Subjects with hyposmia (*N*, %)	5 (50%)	0 (0%)	0.03
*TDI*-score (mean ± SD)	30.1 ± 4.8	34.1 ± 1.9	0.04
*T*-score (mean ± SD)	7.6 ± 1.4	6.8 ± 2.5	0.2
*D*-score (mean ± SD)	9.9 ± 2.8	12.8 ± 2.2	0.02
*I*-score (mean ± SD)	12.6 ± 2.0	14.5 ± 0.7	0.01
